# Primary Intraosseous Cavernous Hemangioma in the Skull

**DOI:** 10.1097/MD.0000000000003069

**Published:** 2016-03-18

**Authors:** Yi Yang, Jian Guan, Wenbin Ma, Yongning Li, Bing Xing, Zuyuan Ren, Changbao Su, Renzhi Wang

**Affiliations:** From the Department of Neurosurgery, Peking Union Medical College Hospital, Chinese Academy of Medical Sciences, Beijing, China.

## Abstract

Primary intraosseous cavernous hemangiomas (PICHs) are benign vascular tumors that may involve any part of the body. PICH occurs more frequently in the spine and less commonly in skull. The earliest description in the English literature was in 1845 by Toynbee, who reported a vascular tumor arising in the confines of the parietal bone. Skull PICHs do not always have typical radiologic features and should always be considered in the differential diagnosis of malignant skull lesions. We now reviewed and analyzed related literatures in detail with reporting a rare case of PICH in the left front bone that was surgically resected.

## INTRODUCTION

**FIGURE 1:**
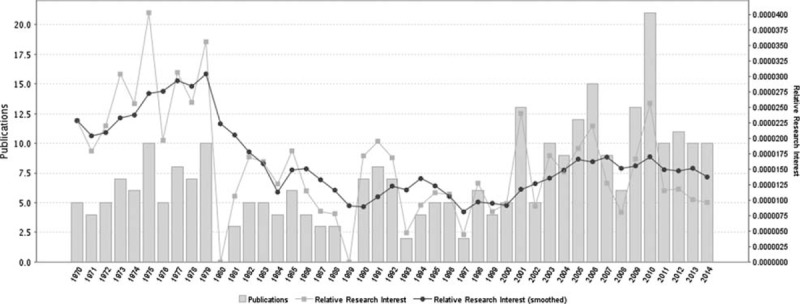
A timeline of the publications related to skull PICH.

**FIGURE 2:**
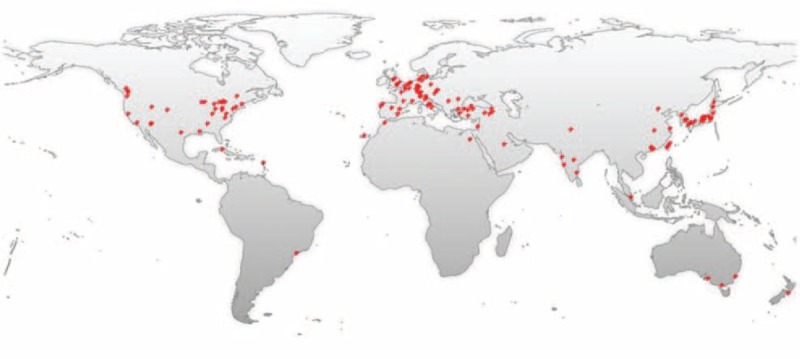
A world map with the global distribution of skull PICH-related publications based on the analysis of their geolocational data.

Primary intraosseous cavernous hemangiomas (PICHs) is a rare bone tumor accounting for 0.7% to 1.0% of all bone tumors.^1^ PICHs are usually found in the vertebral column and rarely seen in the skulls. The earliest description of skull PICH was in 1845 by Toynbee. Much to our knowledge, there have only been 93 cases of skull PICH reported previously. The number of relevant literatures each year shows a general tendency to increase over time. A timeline of the related publications is available as Figure 1. On the basis of a world map with the global distribution of skull PICH-related publications based on the analysis of their geolocational data, the countries that the publications are from are mainly concentrated in Europe, North America, and East Asia (Figure 2).

PICH is mostly seen in middle age and the male-to-female ratio ranges from 3:1 to 2:1.^2^ Among the skulls, the frontal bone is the most commonly involved, followed by the parietal bone, temporal bone, and less frequently by the occipital bone. The pathogenesis is still unknown but a history of trauma seems to be related in some case reports.^3^ Total surgical excision is the treatment of choice and the prognosis after complete excision is excellent and recurrence is usually rare. Herein, we present a rare case of a skull PICH in a 17-year-old girl. The clinical presentation, pathology, differential diagnosis, and treatment of this rare disorder are discussed. Written informed consent was obtained from the patient for publication of this case report and any accompanying images. A copy of the written consent is available for review by the editor of this journal. Because of this, there is no need to conduct special ethic review and the ethical approval is not necessary.

## CLINICAL CASE DESCRIPTION

A 17-year-old girl presented with a swelling on the left forehead, which had been slowly enlarging for 2 year. She denied headache, dizziness, and past trauma history. Other medical history and a review of systems were insignificant. On examination, the diameter of the mass on the left frontal bone was about 6 cm long. In consistency, the lesion was immobile, hard, and not tender. There is no abnormality for routine biochemical tests.

**FIGURE 3:**
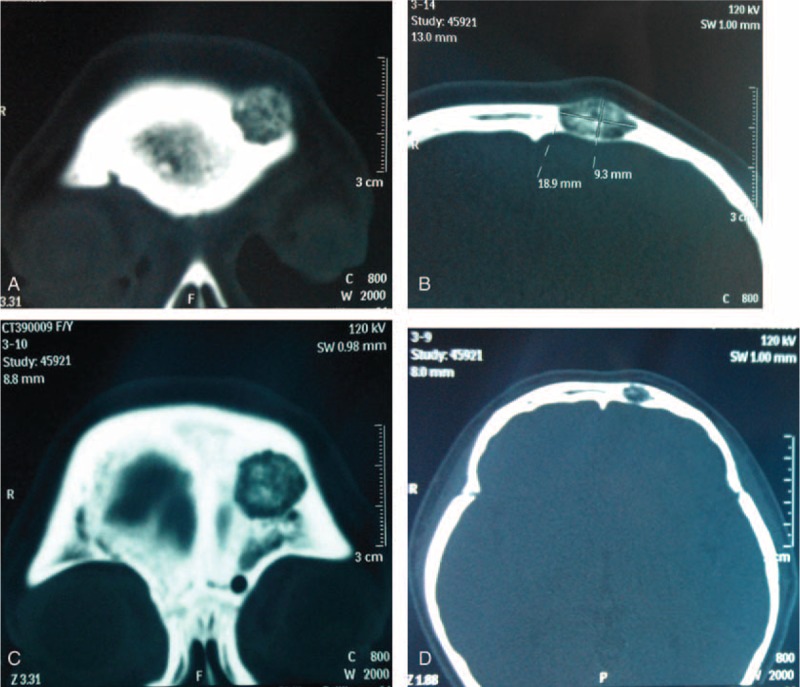
CT scan (bone window) demonstrated an intradiploic osteolytic mass.

Plain cranioaural x-ray demonstrated a radiolucent mass and the CT image showed a 9.3 mm × 18.9 mm × 13.4 mm osteolytic lesion within the diploe on the frontal bone near the orbital roof (Figure 3). Original impression of the mass by a radiologist was a skull eosinophilic granuloma or a fibrous dysplasia. A left parietal craniectomy and total lesion resection with a margin of surrounding normal bones was conducted under general anesthesia. The lesion extended intracranially to the adjacent dura mater loosely and externally to the corresponding scalp. It was rich in blood supply by clusters of small vessels. After surgery, a histologic examination of the specimen demonstrated an introdiploic cavernous hemangioma featured by expanded small blood vessels with thin wall and sinusoids surrounded by a thin layer of endotheliocytes. The patient had a good recovery after surgery. At 1-year follow-up there was not any lesion recurrence.

## DISCUSSION AND REVIEW

**FIGURE 4:**
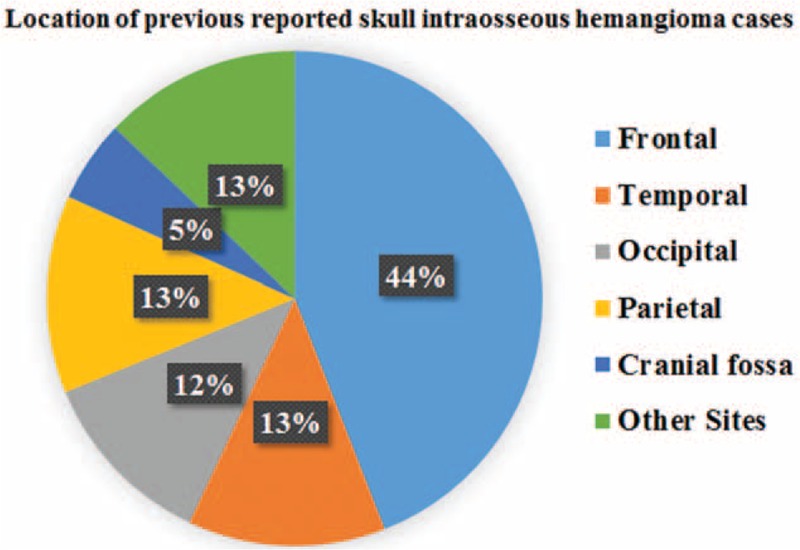
Pie graph of the location of previous reported skull intraosseous hemangioma cases.

Hemangiomas can be histologically divided into 3 types: cavernous, capillary, and mixed. Cavernous hemangioma consists of clusters of dilated blood vessels, which are separated by fibrous septa, whereas capillary ones are rich in small vascular luminas without much fibrous septa. The majority of hemangiomas in skull are of the cavernous type (PICHs), while hemangiomas in vertebras are usually the capillary type. PICHs of the cranium are rare benign vascular tumors that account for about 0.2% of all bone tumors and 10% of benign skull tumors.^3^ It occurs most commonly in the vertebral column and rarely in the skull. Of the 93 cases of skull PICH reported in previous literatures from 1845 to 2015, 44.1% were located in the frontal bone, 12.9% involved the temporal bones, 11.8% occurred in the occipital bone, 12.9% in parietal bone, and 5.4% in Cranial fossa; fewer cases have been reported in sphenoid, zygomatic, ethmoid, clivus, and orbital rim, etc. (Table 1 and Figure 4).

**TABLE 1:**
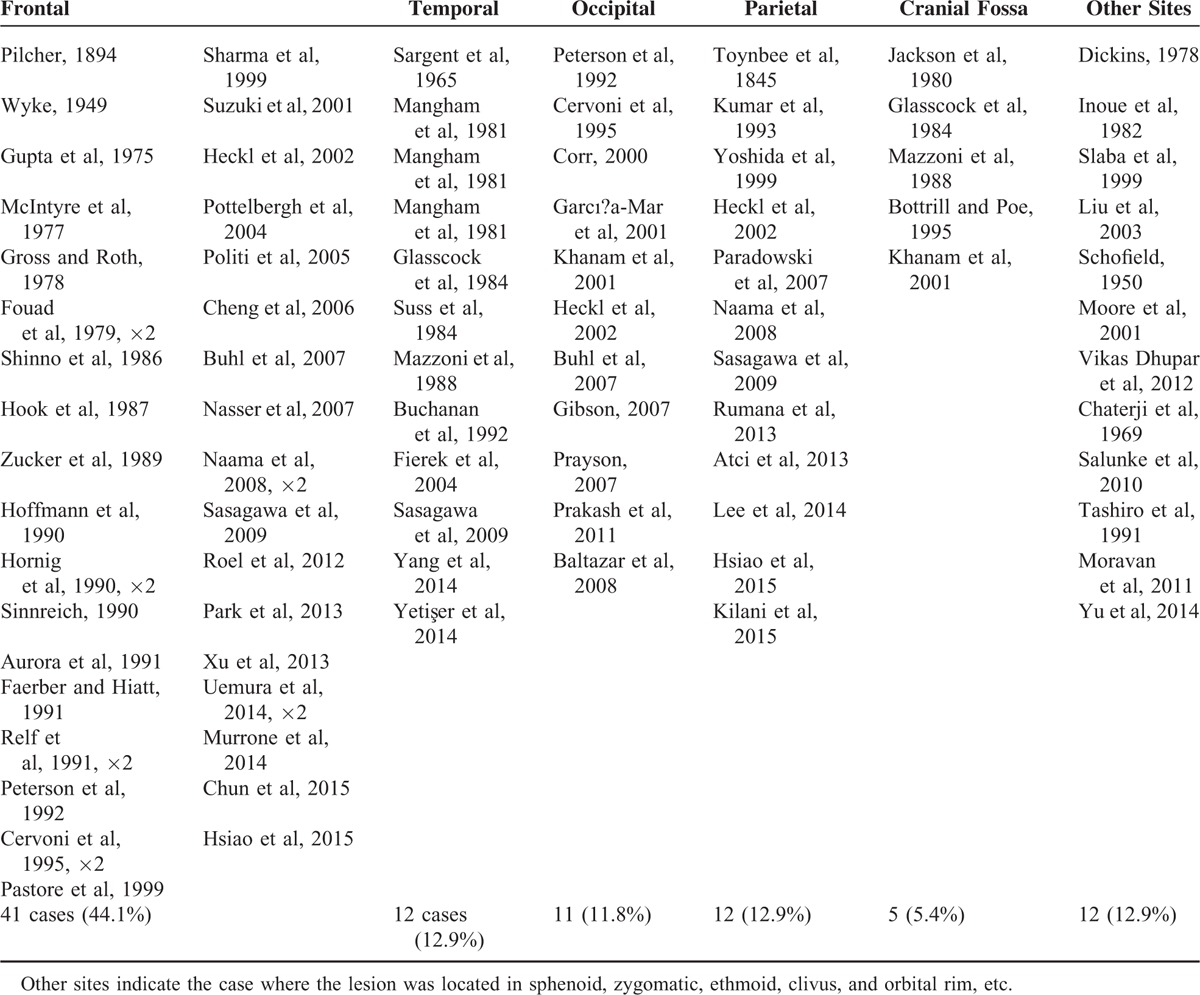
Literature Review of Cases of Intraosseous Hemangioma of the Skull From 1845 to 2015 (Total 93 cases)

They are predominantly seen in patients in their fourth and fifth decades. Unlike the age predominance, our patient was an adolescent female (17-year old). The male-to-female ratio ranges from 3:1 to 2:1.^2^ The earliest description in the English literature was in 1845 by Toynbee, who reported a vascular tumor arising in the confines of the parietal bone.^4^ PICH arises from the vessels in the diploic space and supplied by the branches of the external carotid artery, arising in the skull vault. The middle and superficial temporal arteries are the main sources of blood supply. Within the lesion, the capillaries are widely dilated separated by fibrous tissue. The pathogenesis of PICH remains unknown. The cause is considered to be congenital, but this has not yet been proven. Some scholars proposed a hereditary nature for “vascular malformations” in the skull with an autosomal recessive inheritance mode. Others indicated that proliferation and differentiation of the undifferentiated primitive mesenchymal cells induced by a variety of stimuli may be the potential etiology. Trauma may also be an important etiology.^5^

Like our present case, the patient presented with a slow-developing palpable firm swelling without tenderness. Local neurological deficits were uncommon partly because the masses are more inclined to extend externally than intracranially. A variety of clinical manifestations may occur depending on the involved sites.^6^ Proptosis and impaired vision may appear if the orbit was invaded. Facial nerve paralysis, twitching of oral commissure, pulsatile tinnitus, and hearing loss may occur if temporal bones are involved. Patients may rarely present with an associated epidural hematoma or subarachnoid hemorrhage. We reviewed some previously reported cases of skull PICH since 2000 (Table 2    ).^1–22^ The commonest clinical feature is a solid swelling in the skull, painful or painless. Some patients may also present with headache or dizziness.

**TABLE 2:**
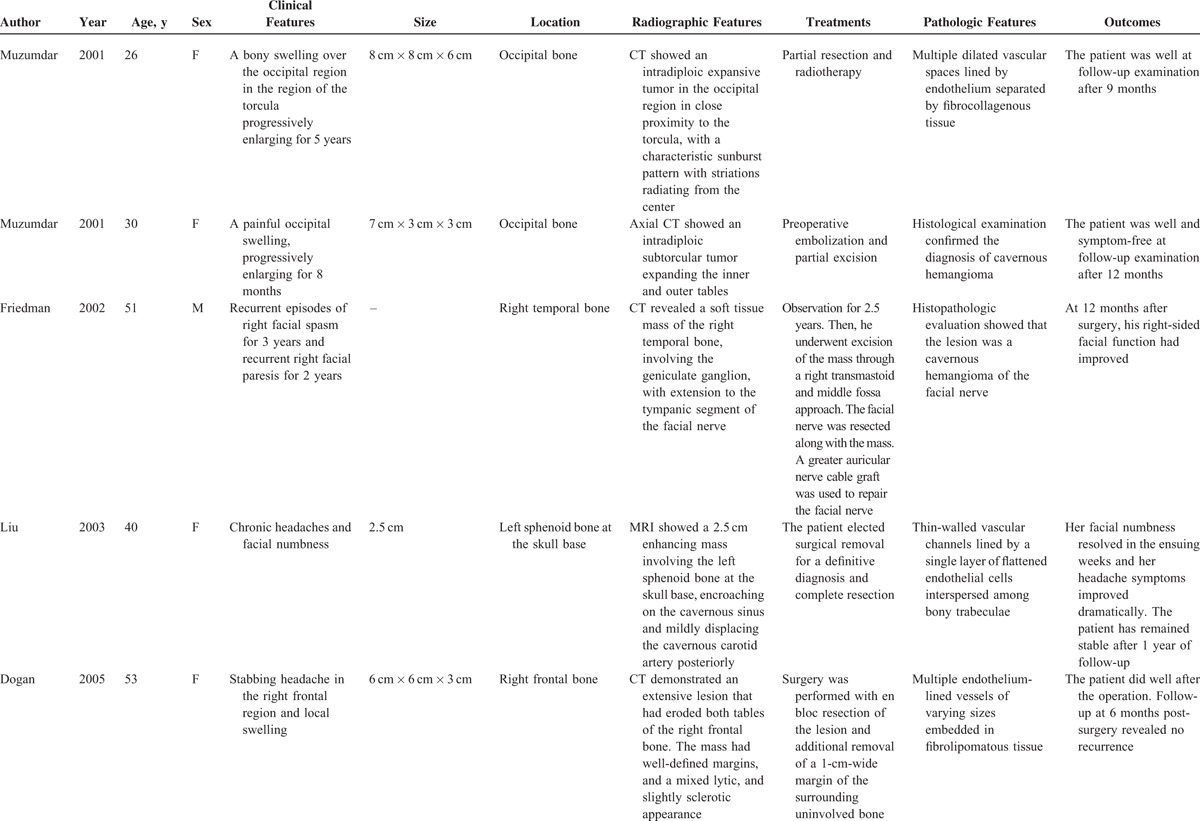
Characteristics of Some Previously Reported Cases of PICH in the Skull Since 2000

**TABLE 2 (Continued):**
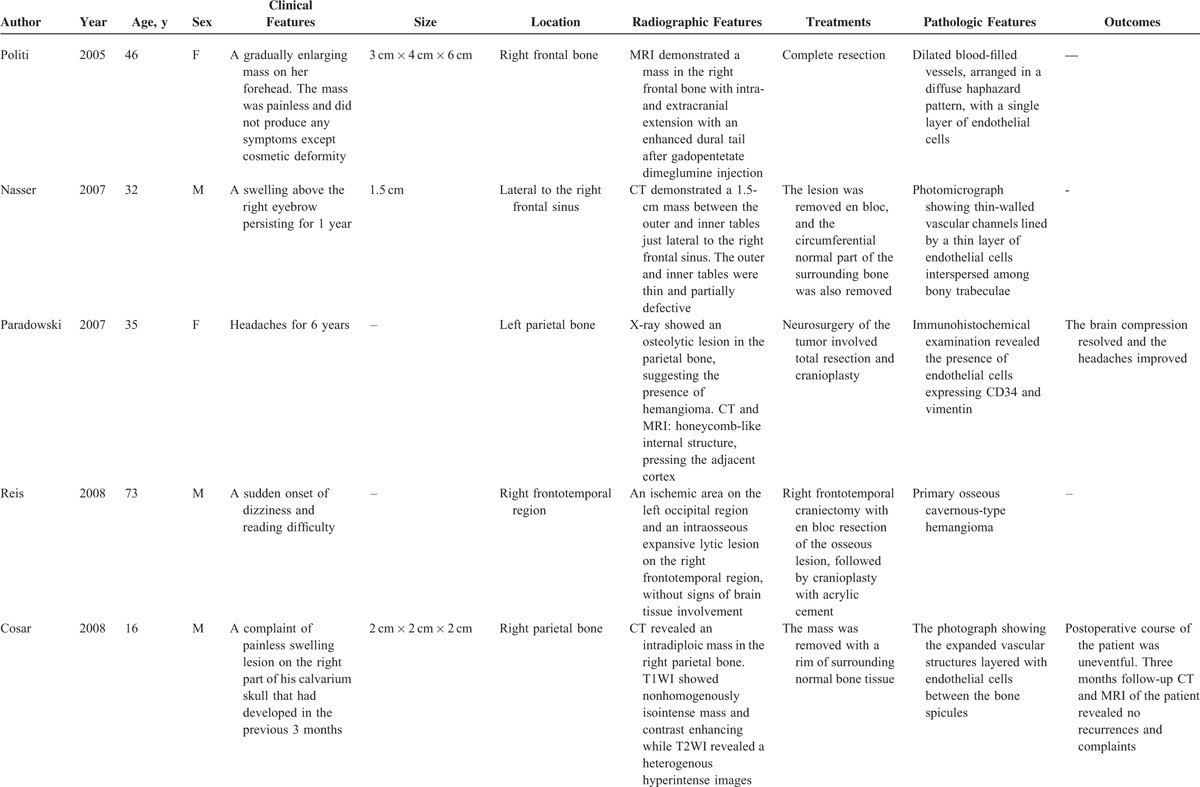
Characteristics of Some Previously Reported Cases of PICH in the Skull Since 2000

**TABLE 2 (Continued):**
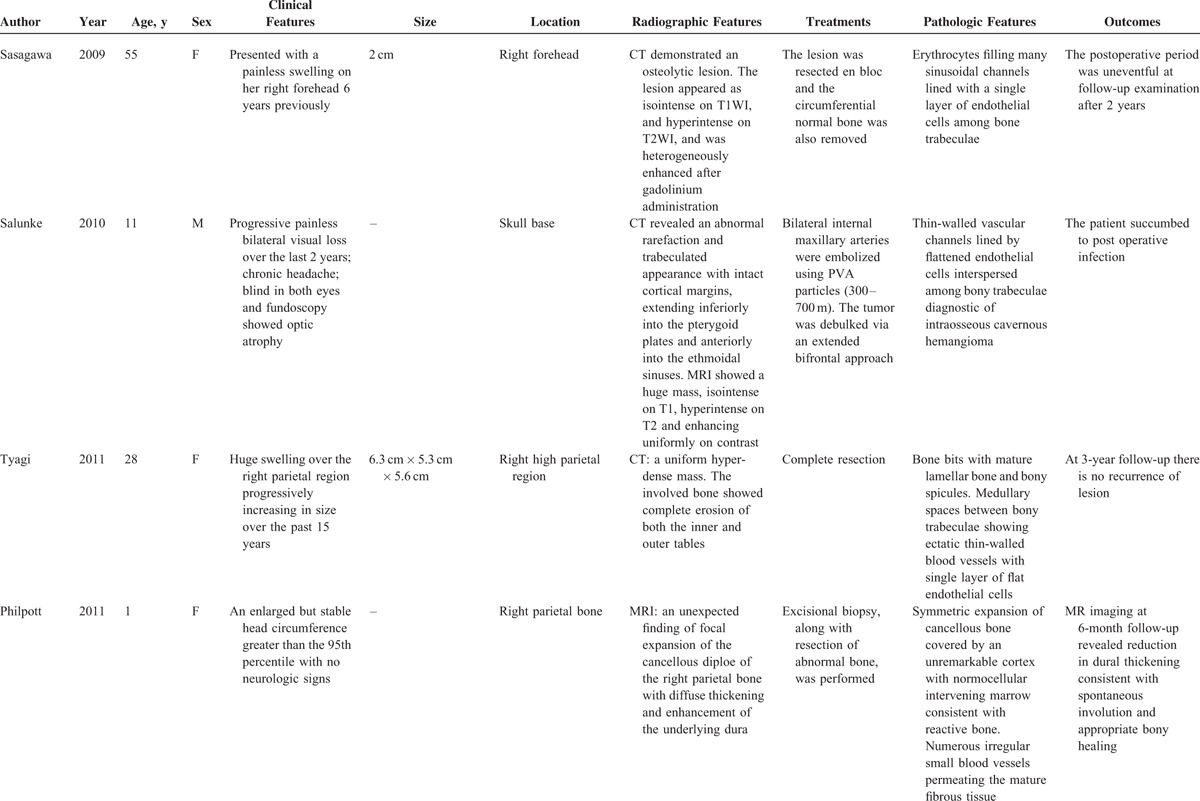
Characteristics of Some Previously Reported Cases of PICH in the Skull Since 2000

**TABLE 2 (Continued):**
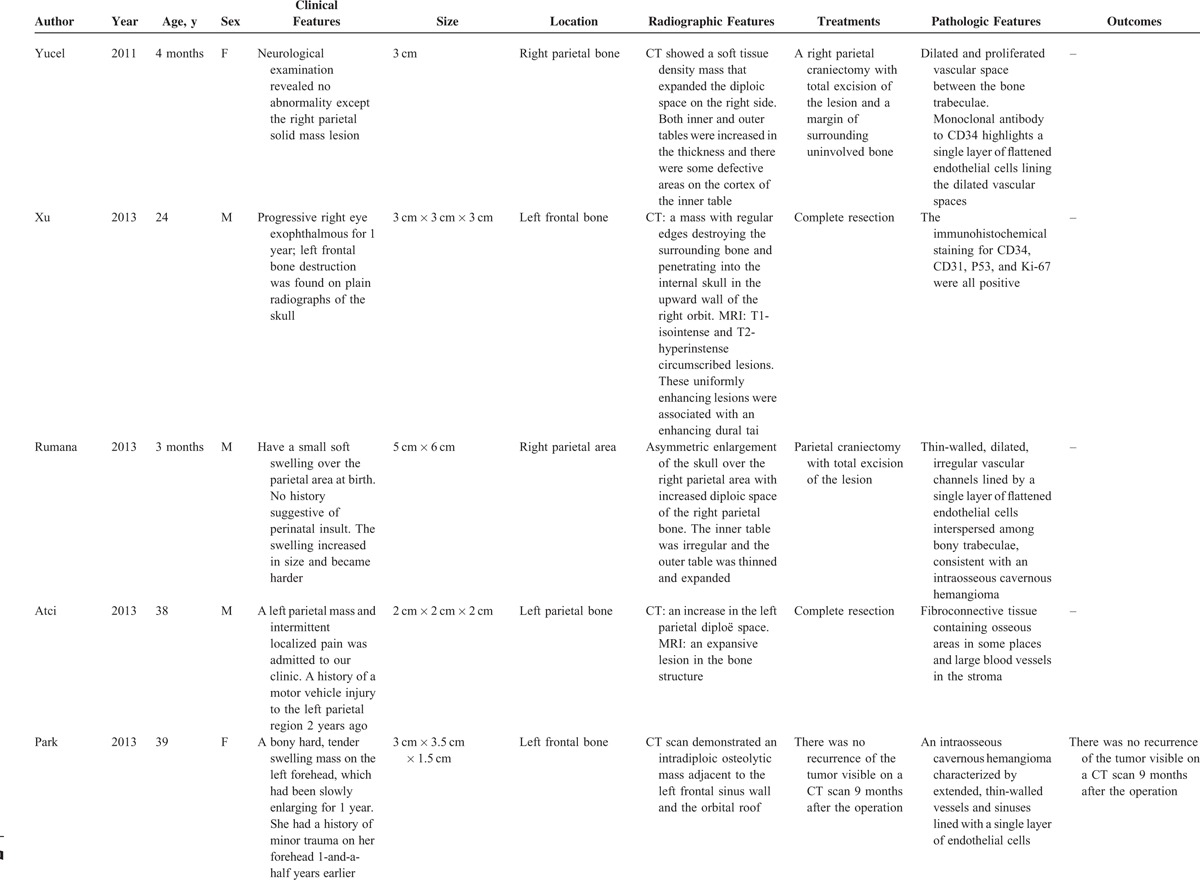
Characteristics of Some Previously Reported Cases of PICH in the Skull Since 2000

**TABLE 2 (Continued):**
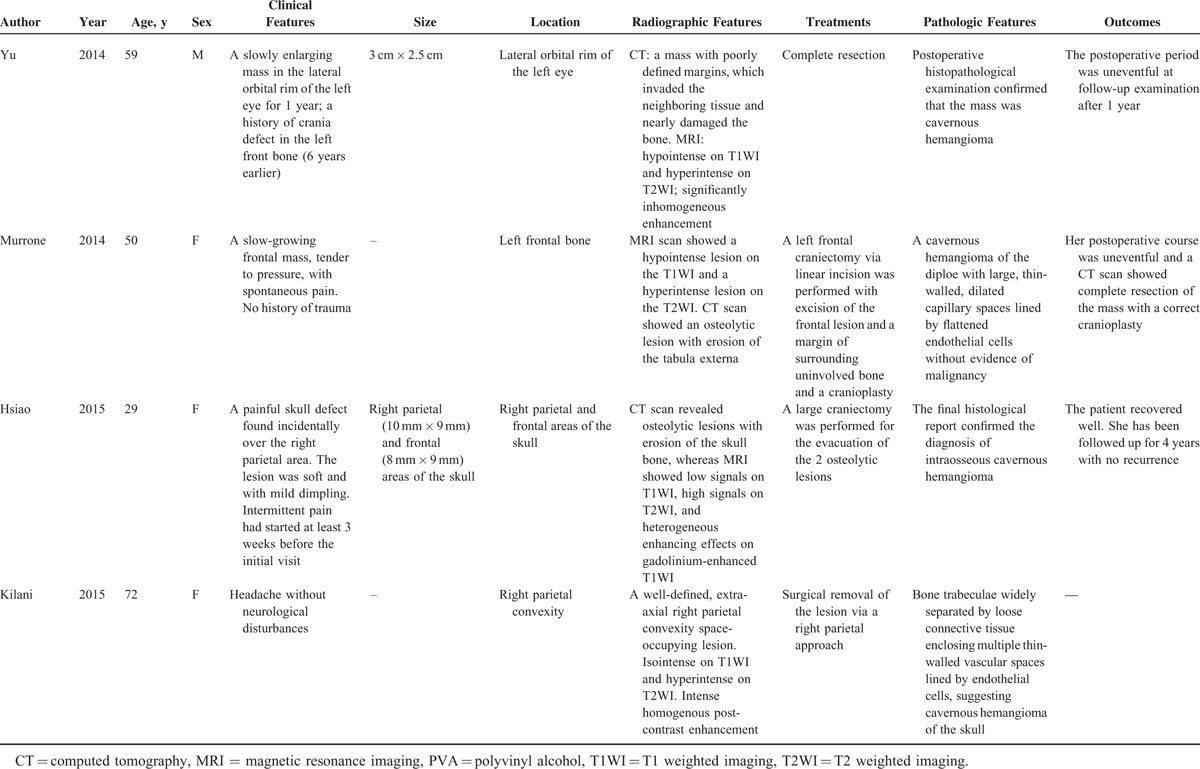
Characteristics of Some Previously Reported Cases of PICH in the Skull Since 2000

Radiologic evaluation includes plain skull x-rays, CT scan, and magnetic resonance imaging (MRI).^9^ CT is an excellent investigation, as it allows detailed characterization of the cortical and trabecular bone to be made. Although the appearance on CT may vary, an expansive lesion with thin borders and intact internal and external skull plates is the most common finding. MRI signal intensity depends on the amount of venous stasis in the lesion and also on the rate of transformation of red marrow into yellow marrow. Although T1WI may give high- or low-intensity signals, water-sensitive sequences, such as T2WI and FLAIR, commonly give high-intensity signals.^6,9^ The CT or MRI features of the cases previously reported are also shown in Table 2    .

The differential diagnosis for intradiploic skull masses includes dermoid tumors, metastatic diseases, meningiomas, sarcomas, Langerhans cell histiocytosis, eosinophilic granulomas, fibrous dysplasia, giant cell tumors, aneurysmal bone cysts, osteomas, Paget diseases, and so on.^4,23^ Because the imaging findings are not specific, preoperative diagnosis is difficult and histopathology is essential. A thorough clinical, radiographic, pathologic, and treatment comparison to other entities in the differential diagnosis is seen in Table 3  .

**TABLE 3:**
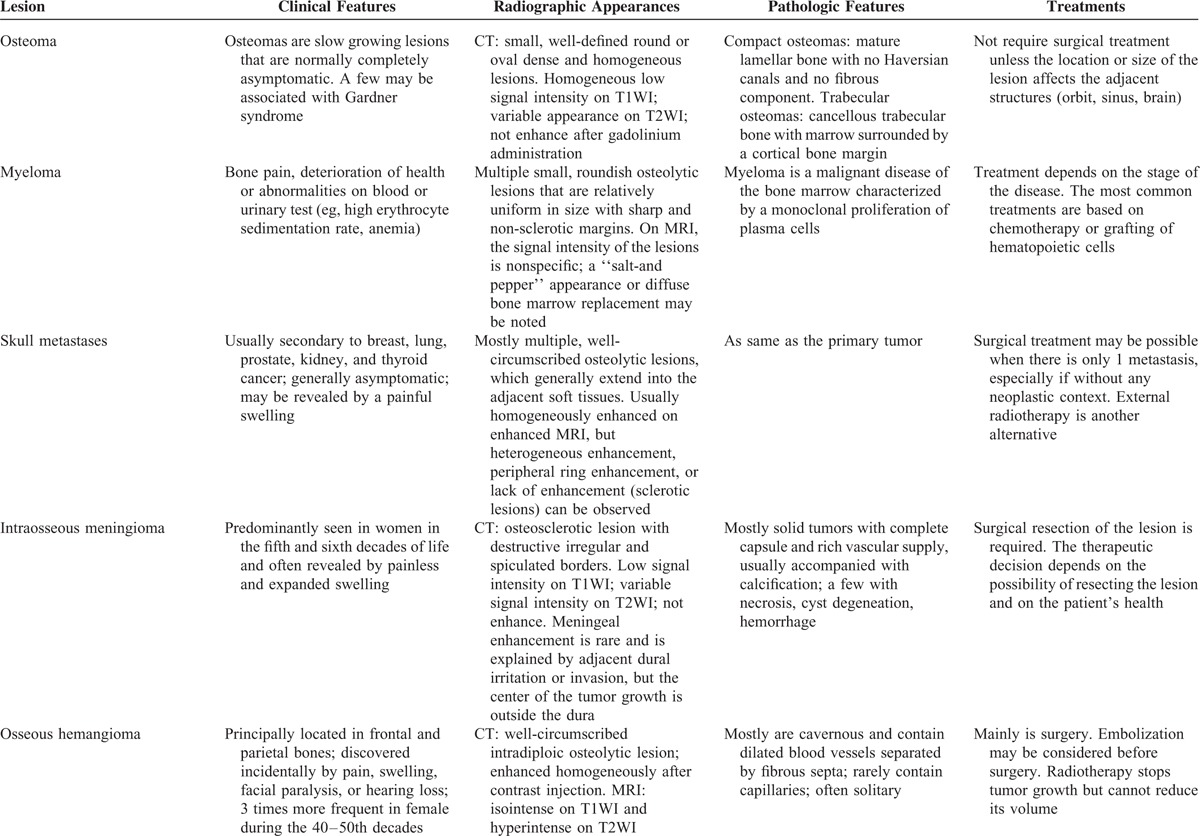
Differential Diagnosis of Skull Vault Lesions

**TABLE 3 (Continued):**
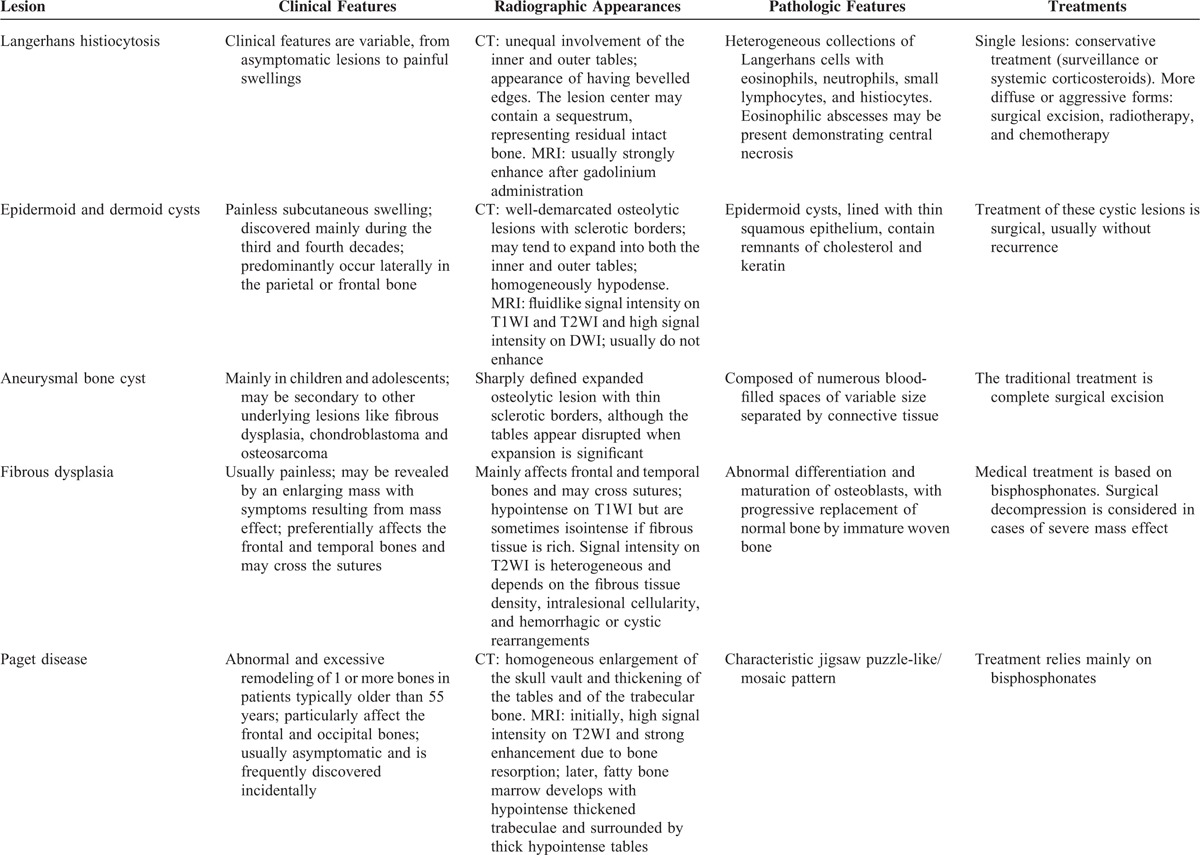
Differential Diagnosis of Skull Vault Lesions

**TABLE 3 (Continued):**
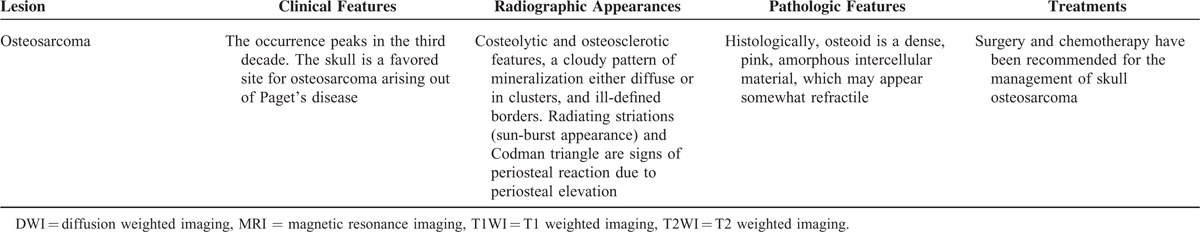
Differential Diagnosis of Skull Vault Lesions

The treatment of choice for skull PICH is total resection with an adequate normal bone margin to reduce the risk of bleeding.^6^ The bony defect can be reconstructed by virtue of a variety of methods.^24^ Relapse is uncommon if sufficient safety margins are achieved. Other treatment options include curettage which can be followed by recanalization and irradiation, which may reduce the tumor volume and has demonstrated symptomatic improvement, but has the risk of radiation-induced carcinoma.^2,4^ Radiotherapy alone can only can prevent the tumor from growing, but it cannot eradicate the lesions. In keeping with the most widely recommended technique, we opted for a craniectomy with total resection, keeping a 0.5-cm safety margin. We then performed cranioplasty with titanium plate.

## CONCLUSIONS

PICHs of the skull are rare benign lesions of vascular origin, showing so semblable medical imaging findings to many other bone lesions that it is hard to differentiate them. Thus, there hemorrhagic features within operative fields and histopathologic examinations remain as the “gold standards” for diagnostics. Total resection with enough uninvolved bone margins must be attempted. PICH's relapse is rare when this surgery is successful.
